# Exploring the Biology of Quasi-Social Idiobiont Parasitoids in the Genus *Sclerodermus* (Hymenoptera: Bethylidae)

**DOI:** 10.3390/insects15110880

**Published:** 2024-11-09

**Authors:** Serena Malabusini, Daniela Lupi

**Affiliations:** Department of Food, Environmental and Nutritional Sciences (DeFENS), University of Milan, 20133 Milan, Italy; serena.malabusini@unimi.it

**Keywords:** offspring production, developmental time, meta-analysis, genus biology

## Abstract

Species in the genus *Sclerodermus* are among the most socially complex parasitoids, unlike most parasitoids, which are solitary and do not provide care after laying eggs. In *Sclerodermus*, groups of females paralyse their host, lay eggs on it, and work together to care for the brood (a quasi-social form of reproduction). This research, through database analysis and meta-analysis, covers the biology of the genus, which has 80 species, though only 24 have been studied in detail. It describes their morphology and behaviour, focusing on offspring production, developmental time, and the factors influencing these, such as kinship and the number of females tending the brood. The materials and methods used provide a comprehensive approach to data collection and analysis, drawing on diverse sources, rigorous classification, and advanced statistical techniques. This approach revealed that *Sclerodermus* species display a high degree of consistency in their responses to temperature, host size, and foundress number.

## Simple Summary

The genus *Sclerodermus* is unique among parasitoid Hymenoptera because it exhibits quasi-social behaviour. It is only in recent years that researchers have started to explore its biology in the laboratory, as it is difficult to observe in the wild, where larvae typically feed on wood-boring hosts. This research aims to provide an overview of the current knowledge of the genus through database analysis and meta-analysis by comparing data from different studies. The comparison of *Sclerodermus*’ biology revealed a high degree of consistency between species in their biological traits.

## 1. Introduction

The family Bethylidae, which belongs to the aculeate Hymenoptera, includes more than 2000 species [1,2,3,4,5,6] and is part of the superfamily Chrysidoidea, whose members are almost all parasitoids [7]. Bethylids are distributed worldwide and feed as ectoparasitoids on host larvae and, more rarely, on the pupae of coleopterans and lepidopterans [8]. Some bethylids are also used as agents of biological pest control [9].

The species in the families can be divided into eight subfamilies: the Pristocerinae Mocsáry, 1881, the Epyrinae Kieffer, 1914; the Mesitiinae Kieffer, 1914; the Bethylinae Haliday, 1839; the Scleroderminae Kieffer, 1914; the Lancepyrinae Azevedo & Azar, 2012, the Protopristocerinae Nagy, 1974, and the Holopsenellinae Engel, Ortega & Azevedo, 2016. The latter three subfamilies are considered extinct, as most of them were detected in amber [4].

The genus *Sclerodermus* (Hymenoptera: Bethylidae) is one of the 13 known genera among the subfamily Scleroderminae and includes at least 80 species with a worldwide distribution [1,2,4], of which 17 are present in the Mediterranean area [10].

Members of the genus *Sclerodermus* are among the most socially complex parasitoid species. In contrast to the vast majority of parasitoid taxa which are socially solitary, and lack post-oviposition parental care [11], *Sclerodermus* is considered a quasi-social parasitoid as its reproduction involves multiple foundress females that share the host and care for offspring communally [12,13,14,15].

The adult size of *Sclerodermus* spp. varies between 1.5 and 6 mm, with the male visibly smaller than the female [16]. Female dimensions vary between and within species (for example: weight: from 0.4 mg to 1.2 mg in *S. guani* [17], from 0.2 mg to 0.8 mg in *S. puparie* [18], from 0.1 mg to 1.0 mg in *S. harmandi* [19]; dimension: from 2.0 to 3.5 mm in *S. puparie* [20]). The body size of female offspring are determined independently from the size of their mothers [20]. This suggests that offspring size is determined by a trade-off between clutch size and the host body size. In many parasitoids, the size of adults is correlated with the size of their larval stage, as the development of eggs and pupae is static [21,22]. The presence of a greater number of offspring feeding on a host leads to increased competition, which in turn leads to an acceleration of larval growth rates. This ultimately results in a potential reduction in adult size [23,24].

It is also noteworthy to mention the polymorphism within *Sclerodermus* species, which is most evident between males and females. Males are darker and smaller than females, but dimensional variability also occurs within the same sex. In addition, polymorphism extends beyond simple size to the absence or presence of wings, including differences in colouration and the presence of ocelli [16]. Winged and wingless individuals occur in both sexes: females are mainly apterous, although micropterous (only in some species, such as *S. domesticus*) and macropterous specimens can be found [16]; males are mainly winged with very few reports of wingless males [25,26,27,28]. Although winglessness affects 75–99.9% of females, higher rates of winglessness occur in larger groups of foundresses [26,29,30,31] or in a foundress subjected to either biotic or abiotic stresses [32]. Among abiotic factors, high temperatures, a short photoperiod and low light intensity positively influence the presence of wings; among biotic factors large brood size and the presence of wingless foundresses can positively influence the presence of wing in some specimens [25]. Colouration can vary from light to dark brown in the females while males are always glossy black [16,25]. The adult has flat eyes with ocelli usually present in macropterous individuals, and normally absent in apterous and micropterous forms. Winged forms are hypothesised to be associated with dispersion [25], as winged parasitoids can reach more distant hosts while apterous ones remain mostly confined to a single tree [33]; the presence of the ocelli in the macropterous specimens but not in the apterous and micropterous specimens supports this hypothesis [16].

The genus *Sclerodermus*, despite exhibiting significant morphological diversity within the same species, is unified by several key characteristics that highlight the complexity and adaptability of its members. All species within this genus share the distinctive behaviour of being parasitising hosts and caring for their offspring, which has facilitated their successful use in biological control programmes. These species are also characterised by a flat, prognathous head, an adaptation that allows them to easily enter the galleries of wood-boring insects to reach their victims.

All the species in the genus share similar biological characteristics and are both idiobiont and sinovigenic parasitoids [33,34,35], as females continue oogenesis throughout adult life [36,37]. As a result, the designated host provides larvae with the resources necessary to complete the development and an environment that can guarantee their survival [18]. Females that choose a victim have to mediate among the difficulty of finding a suitable host, the ability to parasitise it, and the need to keep it clean for the period over which the brood develops, e.g., [19,37,38]. Therefore, the phase of host selection is critical as host quality affects parasitoid fitness, e.g., [11,18,24]. However, larger hosts defend themselves more vigorously than smaller hosts and can inflict damage on parasitoids, e.g., [12,13,14,18,19,39]. Smaller hosts are less aggressive, but their nutritional value may be insufficient to allow the development of the majority of the progeny, resulting in fewer offspring or an increased number of males [18] that require fewer resources to complete the development [23,31]. To summarise, the risk of parasitising smaller hosts is lower than those of parasitising large hosts, but smaller hosts are also more prone to death from desiccation. Both of these scenarios can diminish the chance of effective parasitism [18]. In order to paralyse the hosts, females sting the victim in proximity to intersegmental membranes, which results in the victim rolling over, e.g., in [35]. Although a single female wasp may attempt to parasitise a host, multiple females have been observed to have greater success, particularly with larger hosts. This is thought to be due to their engagement in cooperative host suppression, oviposition and offspring care, e.g., [12,14,26,28,33,40,41].

*Sclerodermus* females may exhibit different types of maternal care to facilitate successful offspring development [35]. Care begins before egg laying as females clean the body of the host by removing detritus [42]; then, after oviposition, they gently and continually pat each egg with their antennae [43]. Additionally, the foundress bites the epidermis at the intersegmental membrane of the host, causing lacunae on the host surface, allowing newly hatched larvae to extend the heads into the body of the victim to suck the haemolymph [35]. Females also use their mandibles and mouthparts to move and clean their offspring. If an egg dislodges from its host, the female picks it up gently with her mandibles and repositions it, e.g., [35,42]. If larvae grow and overlap with the nearest ones, the foundress moves and distributes them on the host surface to limit competition for food and space [35], and if they die, the foundress removes them from the host, e.g., [26,35,43]. Finally, if some of the larvae are infected with fungi or bacteria, the female also removes them from the brood and secretes an antibiotic substance to prevent the development of microorganisms, thus preventing transmission to the remaining offspring [42].

Once the larvae are fully developed and the host has been consumed, the foundresses remove the larvae from it to allow them to spin the cocoon [12,35,42,44]. This is described as a rhythmic translocation behaviour that begins just when the larvae are fully developed [45]. Once the cocoons have been spun, the mothers tend to the brood until the emergence of new adults [12,35,42]. Only after the offspring have emerged can the female search for a new host [28]. The first emergence from the brood is of the males, which, emerging 1 or 2 days before the females, have the opportunity to create a hole in the female cocoons to facilitate mating that generally occurs while the females are just emerged, but still in their cocoon [35].

The primary aim of this article is to explore the similarities, parallelisms, and differences in biology within the genus *Sclerodermus*. To achieve this, we have conducted a comprehensive research and meta-analysis of all available literature on the genus from the earliest records up to the end of 2023, collecting data mainly related to the biological characteristics and lifestyle of these species.

## 2. Materials and Methods

### 2.1. Data Collection

A database with worldwide literature on *Sclerodermus* spp. was created using different databanks: Web of Knowledge, Scopus, Google Scholar, Explora/Minerva (UNIMI Platform), and Research Gate. Additional articles were found in the literature listed in other publications. Conference presentations and proceedings were excluded from the research as they were sometimes difficult to find or uninformative. The studies were not conditioned by the language and all data collected were updated to December 2023.

The following search terms were used: “*Sclerodermus*”, “Bethylidae”, “Behaviour AND *Sclerodermus*”, “Behaviour AND Bethylidae”, “*Sclerodermus* AND development”, “parasitoid AND Bethylidae”, “parasitoid AND *Sclerodermus*”, “Biology AND *Sclerodermus*”, “Morphology AND *Sclerodermus*”, “Biological control AND *Sclerodermus*”, “field release AND *Sclerodermus*”. In addition, a snowball referencing search [46,47,48] was carried out from articles collected from previous results.

When open access publications were available they were directly downloaded; in other cases, they were found in libraries or requested from authors and stored over the years. All books and papers for which at least the abstract was available were included in the database, but data were only downloaded or extrapolated from the full papers for the analysis in the review. Each scientific paper was included in the dataset by adding the following information: the APA citation, the *Sclerodermus* species, the publication date and the type of paper (article, book). In addition, 10 main topics have been identified (Table 1) in order to organise and easily identify each article by its main theme. As each paper may include additional topics, 18 additional topics were selected. A total of 28 words (Table 1) would form a pool of topics, which would then be assigned to each publication in order of importance, up to a maximum of four theme words per publication.

From each paper, data (raw and mean) relating to developmental time, egg laying, number of adults emerged, sex ratio, etc., were extrapolated considering also, if disclosed, the species of *Sclerodermus* studied, the host species, the number of foundresses and host size. Each data entry was also catalogued in a dataset by noting the rearing conditions in which the tests were carried out (temperature, humidity, and photoperiod). When the data were not explicit in the text or in tables, they were obtained from the graphs, using Engauger digitalizer (Version 12.1) app [49].

### 2.2. Statistical Analysis

The data underwent analysis using Generalised Linear Models via R statistical software, Version 4.3.1 [50]. Given that one subset of the data was of a purely descriptive nature, while another contained the mean values, all analyses were weighted according to the number of replicates for each data item. Log-linear analyses, a log-link function, and a quasi-Poisson error structure were used to investigate factors potentially altering integer data (e.g., brood size or number of males generated) [51,52]. Log-linear models were also used to analyse data derived from integers (i.e., offspring production per foundress) [12,13,14,53]. Following logistic and log-linear analyses, the percentage deviance explained (%Dev) was given as a descriptor.

The effect of foundress number on brood production timing was examined through the use of parametric cohort survival analyses (Weibull models with a time-dependent hazard function and a foundress number fitted as a factor) [13,14,51,54]. Every statistical test was two-sided. Significance was determined by removing terms sequentially from initially complex models and aggregating the levels within the factors to create minimal suitable models [51,52,55].

## 3. Results

### 3.1. Literature Analysis

**Figure 1:**
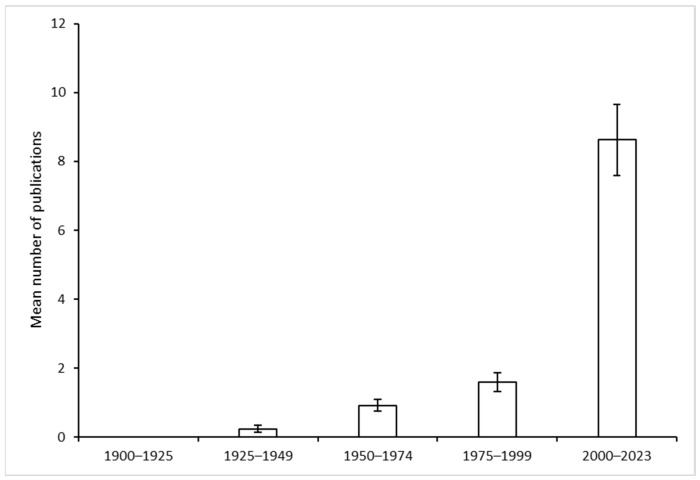
Number of publications per 25 years from 1900 to 2023 (bars indicate standard errors).

A total of 278 articles/chapters were identified, sourced from 134 journals and six books. The study encompasses articles written in eight languages: English, French, Japanese, Chinese, Italian, Korean, German and Spanish. The majority of these papers were published from the 20th century, as the review of literature from the 19th century (from 1800 to 1899) revealed only three articles on this subject [56,57,58]. A total of 68 publications were identified during the course of research conducted in the 20th century. To provide further detail, no publications were identified between the years 1900 and 1924. From 1925 to 1949, six papers were published (2.15% of the total) with an average of 0.24 ± 0.10 per year. Between 1950 and 1974, there were 23 publications (0.92 ± 0.17 per year), and 40 more appeared from 1975 to 1999, peaking in 1985, 1991 and 1999. In the 21st century (2000–2023), the number of articles on *Sclerodermus* species rose significantly, with an average of 8.63 ± 1.03 per year, excluding 2004 when no articles were published (Figure 1).

**Figure 2:**
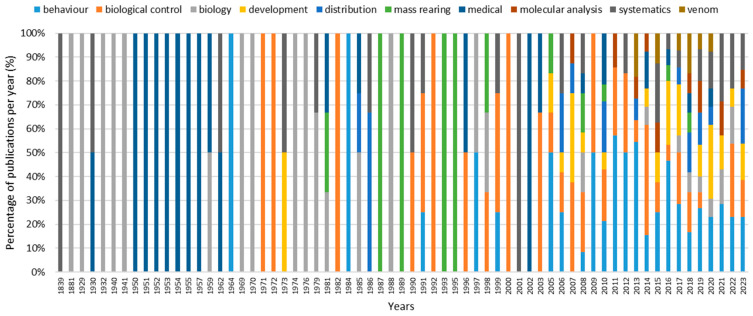
Percentage of main topic per year (each column refers to one single year in which at least one publication was detected).

Figure 2 illustrates the main topics addressed by the different publications over the years. Prior to 1969, the majority of publications focused on biology, systematics and medical issues. Since 1970, the topic of biocontrol has been discussed, and gradually all of the main topics have been covered, with a notable increase in the number of papers dealing with development and behaviour since the 2000s.

In the literature, only 39 species (48.75% of the total) have been studied, and of these, just the following received more detailed attention, being mentioned in multiple papers: *S. abdominalis*, *S. alternatusi* Yang, *S. brevicornis*, *S. cereicollis*, *S. domesticus*, *S. guani* Xiao et Wu (1983), *S. harmandi* (Buysson, 1903), *S. immigrans* Bridwell (1918), *S. macrogaster* Ashmead (1887), *S. nipponicus* Yuasa (1930), *S. pupariae* Yang & Yao in [42], *S. rufescens* Nees (1834), *S. sichuanensis* Xiao (1995) and *S. turkmenicum* Mamaev & Kravchaenko (1973). *Sclerodermus guani*, *S. domesticus*, *S. sichuanensis* and *S. pupariae* cover the majority of the papers and, respectively, 36% (N = 99), 12% (N = 34), 11% (N = 30) and 10% (N = 28) of the cases; moreover, given the difficulty in the classification of the species, the 14% (N = 38) of the sources refers generally to *Sclerodermus* sp. Notice that although *S. guani* and *S. harmandi* are sometimes reported as synonyms [30,59,60], we considered them separately in accordance with the findings of recent research [40,42], which provides evidence that they are in fact different.

**Figure 3:**
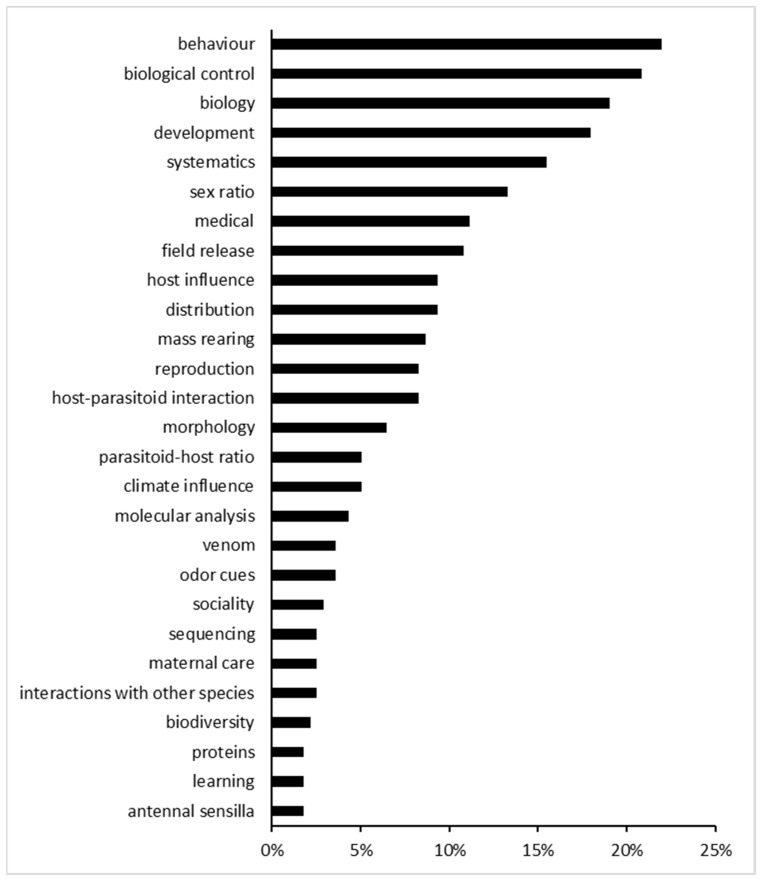
Histogram representing the percentage of the papers in relation to the different topics covered.

Considering all the 28 topic words together, the papers were classified as in Figure 3; as a result, the topic words “behaviour”, “biological control”, and “biology”, covered, respectively, 22% (N = 61), 21% (N = 58) and 19% (N = 53) of the articles, while there were minor reports of “development”, and “systematics” (18% (N = 50), 16% (N = 43)).

The classification of the different studies collected (N = 278), in relation with their main topic, showed that 10% (N = 28) of them were related to the medical field (with the majority them (N = 166 (55%)) concerning *S. domesticus*), 22% (N = 61) were related to the study of behaviour, 19% (N = 53) to biological control, and finally, in order of importance, systematics (N = 31, 11%), biology (N = 29, 10%) and development (N = 27, 10%).

### 3.2. Offspring Production

**Figure 4:**
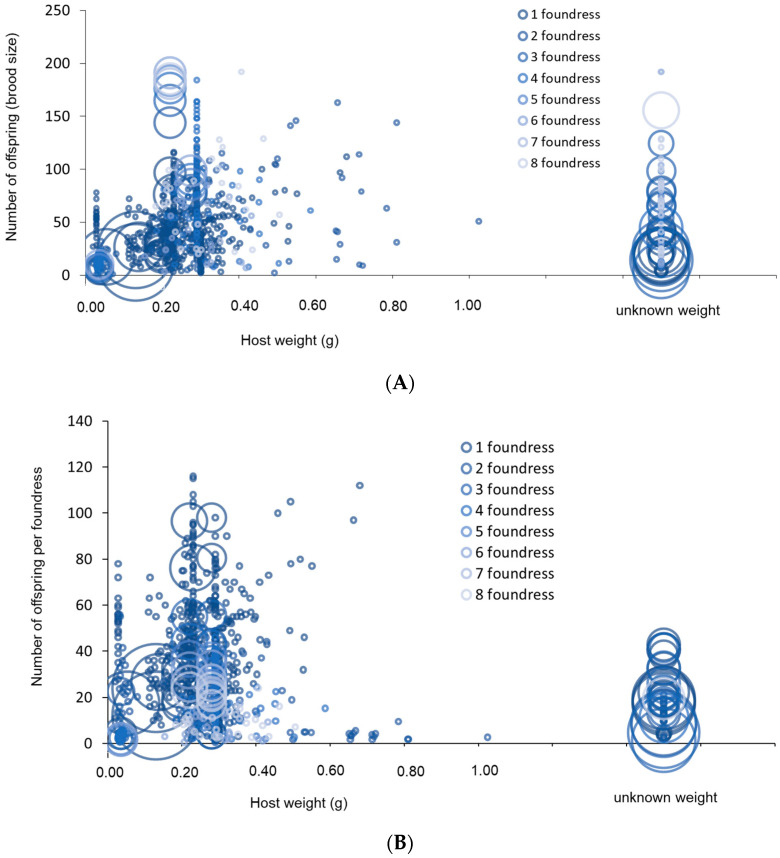
Number of offspring (**A**) and number of offspring per foundress (**B**) as a function of host weight, considering the number of foundresses used. The size of the circle indicates the number of replicates in the paper (when the number of replicates is bigger than 1, the centre of the circle indicates the mean).

A total of 27 publications were classified according to offspring number, foundress number and host size. Figure 4 illustrates the distribution of offspring (pure data and means) obtained from the above-mentioned papers, classified according to host weight and foundress number. Eight papers [29,33,34,41,61,62,63,64] in which the host weight was either absent, or, if present, characterised by a high degree of variability among specimens, were included in Figure 4 as ‘unknown weight’. The largest brood size data resulted in 192 specimens per host when eight foundresses attacked a large host (≃0.4 g) [12]. A mean (±SE) of 243.88 ± 43.14 offspring found was obtained for *S. brevicornis* on *Anoplophora glabripennis* (Motschulsky) (Coleoptera: Cerambycidae) (this value is not present in Figure 4 as there are no data related to the number of replicates) [33]. Finally, the maximum number of offspring obtained when just one foundress was present was 116 [65].

There was a positive correlation between brood size and host size (Pearson’s product-moment correlation: t = 9.9761, df = 889, *p* < 0.001, cor = 0.31) as suggested also by different authors [12,13,14,19,66]. Likewise, the number of offspring per foundress and host size was positively correlated (Pearson’s product-moment correlation: t = 7.2043, df = 907, *p* < 0.001, cor = 0.233). The brood size and number of offspring per foundress had a significantly curvilinear response to the increase in host weight (brood size: log-linear regression including a quadratic term: host weight: F_1, 889_ = 125.20, *p* < 0.001, (host weight)^2^: F_1, 888_ = 113.36, *p* < 0.001, %Dev = 25.85; number of offspring per foundress: log-linear regression including a quadratic term: host weight: F_1, 957_ = 96.47, *p* < 0.001, (host weight)^2^: F_1, 956_ = 204.31, *p* < 0.001, %Dev = 24.91).

**Figure 5:**
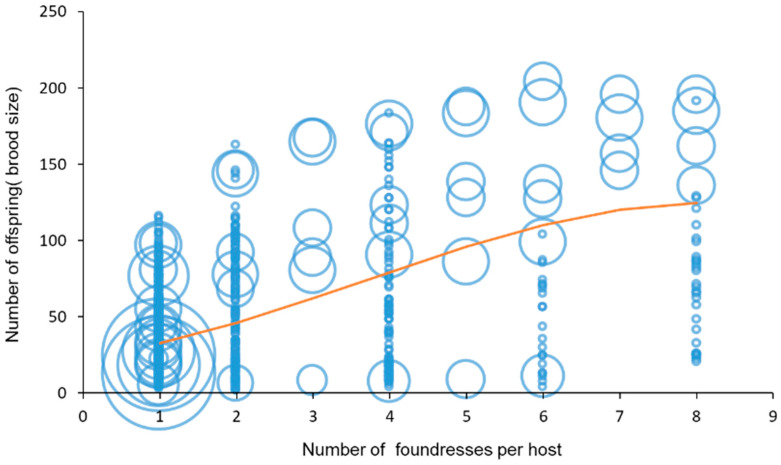
Number of offspring as a function of number of foundresses used. The size of the circle indicates the number of replicates in the paper (when the number of replicates is bigger than 1, the centre of the circle indicates the mean).

As already stated by several authors [12,67], the number of offspring (brood size) is influenced by the number of foundresses according to a curvilinear relationship (log-linear regression including a quadratic term: number of foundresses: F_1, 889_ = 409.88, *p* < 0.001, number of foundresses^2^: F_1, 888_ = 22.719, *p* < 0.001, %Dev = 32.36) (Figure 5).

**Figure 6:**
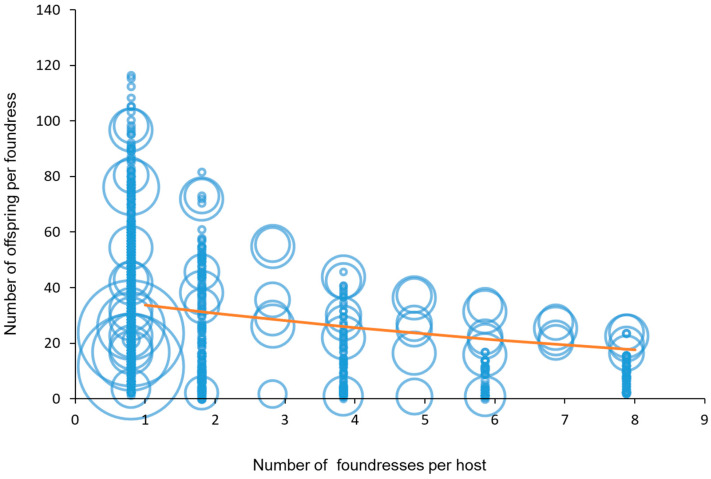
Number of offspring per foundress as a function of number of foundresses used. The size of the circle indicates the number of replicates in the paper (when the number of replicates is bigger than 1, the centre of the circle indicates the mean).

The monotonic decline in Figure 6 is similar to the classical pattern observed in studies of clutch size in gregarious parasitoids [11,68,69,70,71], as per foundress production (offspring/foundress group size) decreased significantly as the number of foundresses increased (F_1, 883_ = 73.03, *p* < 0.001, %Dev = 18.17), as also in [14].

There is only paper that considers the numbers of foundresses to be greater than eight [72]. This paper observes that once the maximum number of foundresses (N = 25) has been reached for the host size considered (large hosts), the total brood per host begins to decline. This is probably due to the emergence of competition and sabotage between the foundresses at higher densities.

The topic “sex ratio” was identified in 14% of the papers (N = 38). As a result in *Sclerodermus* spp., the overall weighted mean, calculated from the analysis of all the papers, was 0.079 ± 0.005, indicating a pronounced female bias in the sex ratio, typically around 0.1 (10% of offspring are males), which is anomalous in comparison to the other Hymenoptera parasitoid species with gregarious offspring, where at least 5% of offspring are males [11]. This could be explained by the local mate competition (LMC) model [73], which suggests that as the number of foundresses increases, the sex ratio should approach 0.5. This is in line with prior models that assume mating occurs throughout the population (panmixia) rather than exclusively within natal groups [74,75,76]. A potential explanation for this strikingly balanced sex ratio has been put forth by several researchers [12,77]. Nevertheless, despite these efforts, a definitive explanation remains elusive. Thanks to current evidence [72,78], the most recent and well-supported hypothesis suggests that dominance or infanticide may underlie this pronounced and observable sex ratio bias [79].

### 3.3. Developmental Time

The key word “development” was associated with 50 papers. To analyse the developmental time, data from 18 of the articles were used (excluded papers did not contain data that could be analysed and plotted).

Given the known influence of temperature on development [65,80,81,82] and the temperature range of 20 °C to 33 °C in the papers under consideration, we investigated the role of temperature in the developmental time of female *Sclerodermus*.

Temperature had a significant impact on the total developmental time of the females, regardless of the species studied (detailed in Table 2, (F_8, 516_ = 155, *p* < 0.001)). It is important to note that the majority of the species included in these studies are of Chinese origin, with the exception of *S. brevicornis*. In accordance with the expected outcomes, an increase in temperature has been associated with a reduction in developmental time. However, as evidenced by the data presented in Table 2, there are some instances where this trend does not appear to align with the expected pattern. This is likely due to the impact of the variability (e.g., ±5, ±10) in the papers considered, which has the potential to influence the analysis when there is a limited number of studies and replicates. This is the reason for the exclusion from the analysis of some experiments at 27 °C (in detail: [83,84]) that were statistically different from others at the same temperature (F_1, 8_ = 87.79, *p* < 0.001). Overall, the fastest development (weighted by number of replicates mean (±SE)) was observed at 30 °C (21.8 ± 0.4 days) and 33 °C (22.5 ± 0.94 days) [25,65,82,85], followed by 27° and 26 °C. Finally, the slowest developmental times were at 21 °C (54.5 ± 0.94 days), 20 °C (36.1 ± 1.04 days) and 24 °C (39.1 ± 0.90 days) [8,25,64,85].

In the papers considered, the total developmental time (from egg to adult) of the females was observed in a total of 25 different hosts, of which 4 were the most commonly studied: *Monochamus alternatus* Hope (Coleoptera: Cerambycidae), which was the subject of seven papers: [19,34,38,61,86,87,89]; *Thyestilla gebleri* (Fald.) (Coleoptera: Cerambycidae) [20,65,77,82,83,84,90]; followed by *Psacothea hilaris* (Pascoe) (Coleoptera: Cerambycidae) [14,28,33,61]; and *Tenebrio molitor* L. (Coleoptera: Tenebrionidae) [63,88,91]. Conversely, only six species of *Sclerodermus* were evaluated (*S. alternatusi*, *S. brevicornis*, *S. guani*, *S. harmandi*, *S. puparia* and *S. sichuanensis*). The developmental time of females exhibited significant variation in response to both host species (cohort survival analysis weighted with number of replicates: χ^2^_22_ = 882.01, *p* < 0.001, n = 529) and *Sclerodermus* species (cohort survival analysis weighted with number of replicates: χ^2^_7_ = 660.11, *p* < 0.001, n = 5290). Furthermore, the interaction between these factors and temperature was also significant (host species × temperature: χ^2^_206_ = 4788.11, *p* < 0.001; *Sclerodermus* speciestemperature: χ^2^_71_ = 3293.28, *p* < 0.001).

Considering only the data from papers with a temperature of 25 °C (the largest numbers [746 replicates] are derived from 11 papers), both the host species and the *Sclerodermus* species influenced the developmental time (host species: χ^2^_6_ = 564.57, *p* < 0.001; *Sclerodermus* species: χ^2^_4_ = 684.41, *p* < 0.001). *Sclerodermus* spp. had a faster mean development on *Psacothea hilaris hilaris* (21.2 ± 1.90 days), while the slowest mean development was on *Semanotus sinauster* Gressit (Coleoptera: Cerambycidae) (34.4 ± 0.42 days). The species that completed development the fastest were *S. brevicornis* with a mean time of 24.7 ± 0.39 days [33] and the minimum on one replicate in *A. chinensis* of 13.7 days [33]. In contrast, *S. alternatusi* (35.3 ± 0.34 days) [85] and *S. sichuanensis* (34.3 ± 0.24 days) [88] developed more slowly. The same information is obtained pooling data together, independently from the temperature of rearing, as the interaction between host and *Sclerodermus* species was significant (F_24, 490_ = 11.09, *p* < 0.001) (Table 3). Finally, the fastest host–parasitoid combination to complete the development was *S. puparie* on *Agrilus planipennis* Fairmaire (Coleoptera: Buprestidae) (25.00 ± 0.74 days) [data at 25 °C and 30 °C].

**Figure 7:**
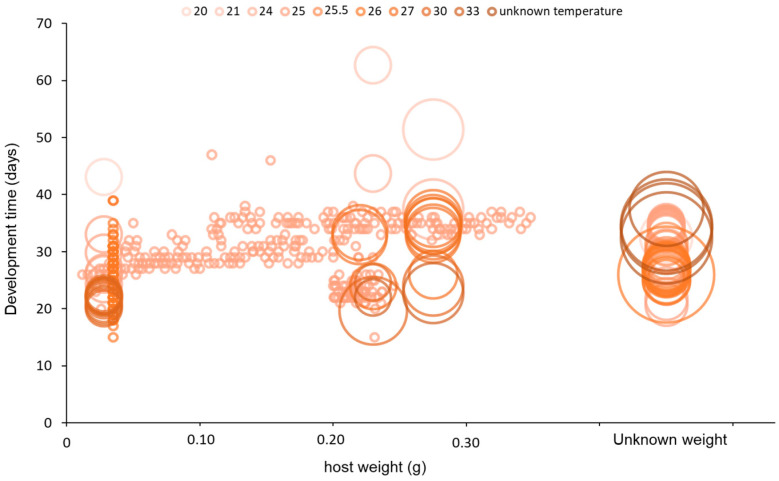
Days taken by *Sclerodermus* spp. females to develop (from egg to adult) as a function of host weight. The size of the circle indicates the number of replicates in the paper (when the number of replicates is bigger than 1, the centre of the circle indicates the mean). The different colours of the circle show the different temperatures considered (most of the papers consider 25 °C). The red line represents the regression line between weight and timing.

The host size also had a significant effect on the total developmental time of females (from egg to adult) (F_1, 475_ = 77.95, *p* < 0.001), as also found by [14]. Figure 7 graphically shows the distribution of the values used for the analysis in relation to the weight (in grammes) and also those tests where the weight and the class were unknown (also taking in account temperature). Several authors explained that the longer development on larger larvae could be related to the fact that they are idiobionts that can immediately use the host, which when large, allows a longer developmental time by providing more reserves [14,92]. Thus, it appears in this case that parasitoids developing on large hosts promote parasitoid body size over the speed of growth [24].

**Figure 8:**
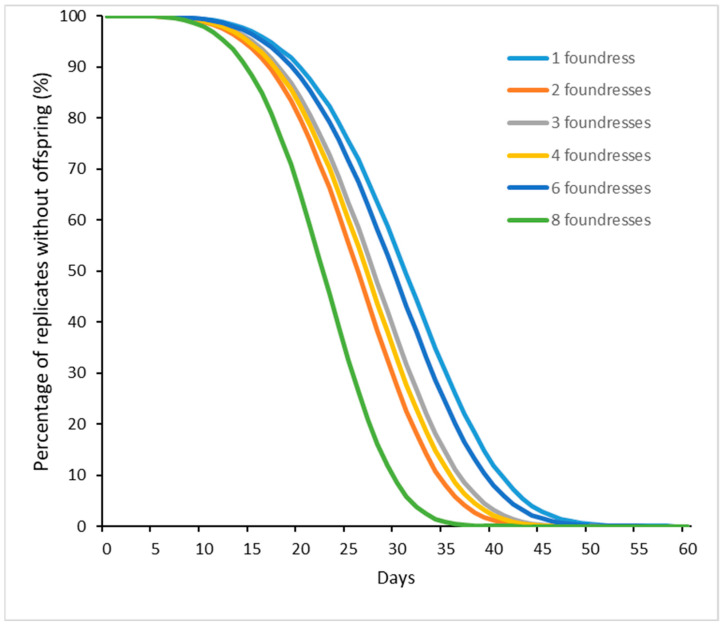
Cohort survival analysis of *Sclerodermus* spp. brood production: Weibull model of offspring production following egg oviposition. Separate relationships are shown for each group of foundress numbers. The difference across these was significant (see main text).

The number of female foundresses was also taken in consideration; this ranged from one to eight foundresses and in some cases more than one host. A significant difference in the developmental time according to the number of foundress females was noted (χ^2^_6_ = 201.81, *p* < 0.001), independently of the temperatures of rearing (Figure 8).

According to [63], the duration of development diminished as the number of parasitoids increased. Furthermore, [13] assessed that the time from oviposition to the emergence of offspring was affected not only by the foundress number but also by the foundress kinship. On the contrary, several authors found no differences in the timing of any developmental stages of *S. guani* [89,90,93] with the exception of the larval stage, but they associated this with parental care [94].

Females always emerged a few days after the males [35], with a slightly longer developmental time. Indeed, analysing the weighted by replicates mean time, independently from the rearing temperature, there is a significant difference in the developmental time of males (27.6 ± 0.63 days) and females (30.0 ± 0.64 days) (F_1, 741_ = 11.66; *p* < 0.001)). Eight papers [18,20,34,60,65,82,83,90] evaluated the length of male developmental time from egg to adult on an average (not weighed) of 25.36 ± 0.31 days (Table 4).

Temperatures influence the timing of male development (cohort survival analysis weighted with number of replicates: χ^2^_6_ = 1175.76, *p* < 0.001). Also, without taking temperature into account, host species seem to influence the developmental time in males (cohort survival analysis weighted with number of replicates: χ^2^_2_ = 98.98, *p* < 0.001), with a difference found between *Massicus raddei*, *Saperda populnea* and *Thyestilla gebleri*, but the data are insufficient to make general considerations on this aspect considering only one temperature. Other authors found that the total timing of development in males was related to host species and stage, and that male development was faster for *S. harmandi* on pupae of *M. alternatus* (vs. *M. saltuarius* and *P. hilaris*) [61].

**Figure 9:**
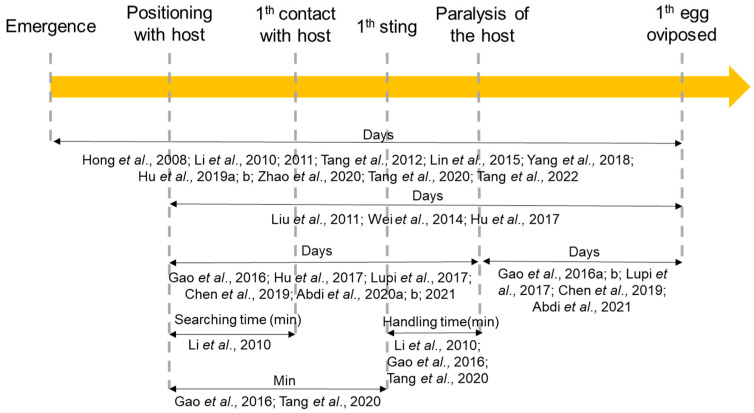
Scheme to summarise the pre-ovipositional time analysed by different authors. (References: [13,14,15,18,19,20,29,33,34,38,61,63,65,82,83,85,87,89,90,91,95,96]).

The pre-ovipositional period has been analysed in several papers, but comparisons are difficult as different authors considered different pre-ovipositional events (Figure 9).

Eleven papers reported the pre-ovipositional period (from emergence to first oviposition), with an overall weighted (by replicates) mean time of 8.91 ± 0.17 days and a significant effect of temperature (F_7, 175_ = 27.37, *p* < 0.001). The details of timing at different temperatures are reported in Table 5.

**Figure 10:**
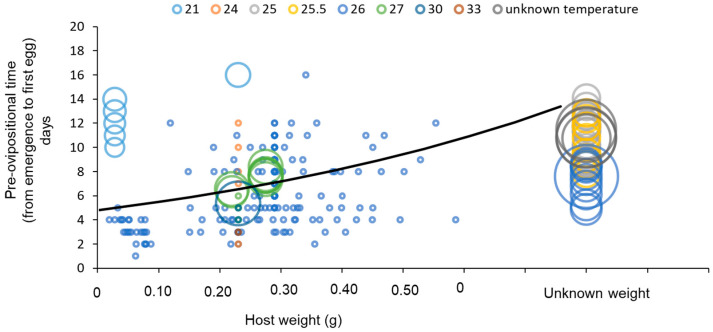
Days taken by *Sclerodermus* females to oviposit as a function of host weight. The size of the circle indicates the number of replicates in the paper (when the number of replicates is bigger than 1, the centre of the circle indicates the mean). The different colours of the circle show the different temperatures considered (most of the papers consider 26 °C). The black line represents the regression line between weight and timing.

**Figure 11:**
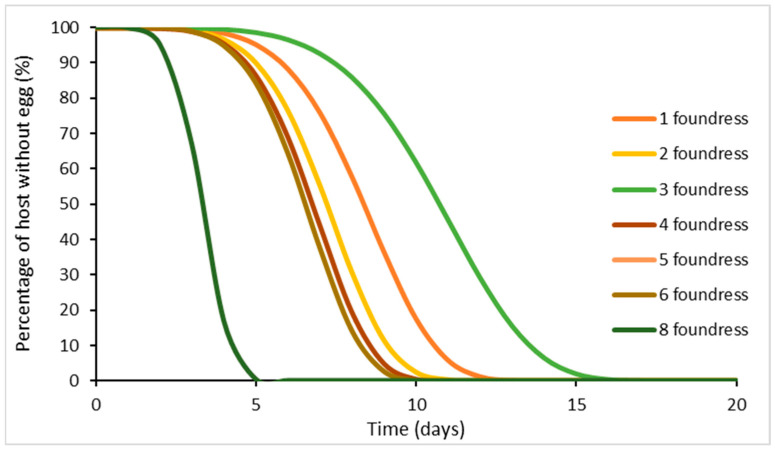
Cohort survival analysis of *Sclerodermus* spp. egg oviposition: Weibull model of egg oviposition following the foundress emergence. Separate relationships are shown for each group of foundress numbers. The difference across these curves was significant (see main text).

In addition to temperature, data indicate that this timing is also significantly influenced by the weight of the host (log-linear regression: F_1, 2489_ = 27.96, *p* < 0.001) (Figure 10) and also by the number of foundresses (cohort survival analysis, weighted with number of replicates: χ^2^_7_ = 909.1, *p* < 0.001) (Figure 11). As the number of foundresses increases, the pre-oviposition period also decreases in a manner consistent with the findings of several authors [89,90,95].

The time between the presentation of the host and the first egg laid has been considered in several articles [18,19,91]. The overall (weighted by replicates) mean (±SE) time for this period was 5.24 ± 0.10 days, but they also reported that the time increased as host size increased [18,19]. This was influenced by temperature (χ^2^_2_ = 57.89, *p* < 0.001), despite the three temperatures considered in the papers displaying only slight variations: 25 °C in [18]; 25.5 °C in [19]; and 26 °C in [91].

Five papers (Figure 9) reported the time between the paralysis and the first oviposition (weighted by replicates mean (±SE): 5.86 ± 0.16 days). Several authors confirmed the reduction in the time period as the number of female foundresses increased, as in the previous cases [15,90].

The weighted mean (±SE) time between the presentation of the host and its paralysis was 5.34 ± 0.22 days according to the available studies [13,14,15,20,33,85,91].

Moreover, only one article [38] considered the searching time (from initial positioning at the host to the first contact with it) as a factor to evaluate the adaptation of *S. harmandi* to a novel host. In this paper, it was observed that after 12 generations, the searching time had decreased (mean value (±SE): 22.15 ± 7.82 min). Additionally, some authors [83,90] also analysed the time (in minutes) between inoculation (positioning) and the first sting, and from these data it was possible to estimate a weighted mean (±SE) of 248.84 ± 63.73 min. This time period was also observed to decrease as the number of foundresses increased (1913 min [one foundress] vs. 376 min [three foundresses] up to a maximum of three; then, it remained stable (overall mean (±SE): 456.38 ± 77.83 min) [90]. Finally, three papers [38,83,90] considered the minutes between the first sting and the paralysis (Handling time), observing a reduction in the time after 12 generations on a novel host [38] and a significant decrease as the parasitoid density increased [90].

## 4. Discussion

The methodology employed in this study for the compilation of a comprehensive study on *Sclerodermus* utilised multiple databases (Web of Knowledge, Scopus, Google Scholar, etc.) and included literature from various countries, thereby ensuring a global perspective. This approach mitigates the potential for bias that could arise from language barriers, thus facilitating a more inclusive review of the scientific contributions on *Sclerodermus* across different regions and scientific communities. Furthermore, the snowball referencing technique adds another layer of thoroughness, capturing literature that may have been overlooked in primary database searches. A noteworthy exclusion was that of conference presentations and proceedings, which are frequently more challenging to access and may contain preliminary data of a less detailed nature. This guarantees the utilisation of superior-quality, peer-reviewed sources while concentrating on published books and papers, thus ensuring a more reliable dataset. The categorisation of papers into ten main topics, supplemented by 18 additional-topics, highlights a structured approach to the organisation of an extensive body of information. This classification system facilitates analysis and highlights the versatility of research on *Sclerodermus* spp. across various scientific domains, including biology and biological control. Furthermore, the restriction of the number of topic words to four per paper ensures that the focus remains on the core contributions of each publication.

By accounting for climatic conditions, the study acknowledges the ecological variability that can influence *Sclerodermus* development, thereby ensuring a more precise data interpretation. The utilisation of digital tools, such as the Engauger digitalizer (Version 12.1) for the extraction of data from graphs, is an example of how modern technology can overcome common challenges in the literature reviews, particularly when data are not readily available in tabular form [97].

The search yielded evidence indicating a considerable increase in the number of articles published on the *Sclerodermus* species over the past two decades. This could be attributed to the growing interest in China first [98,99,100], but also in Europe [15,33], where these species are being considered for applicative uses as biocontrol agents of invasive longhorn beetles. Consequently, the subject matter has undergone a notable transformation in recent years. While the focus was previously centred on biological aspects, it has recently shifted towards the evaluation of behavioural aspects in relation to the use of the *Sclerodermus* species as biological control agents. Nevertheless, despite the considerable number of papers and chapters that have been collated, only 48.75% of the existing *Sclerodermus* species have been subjected to detailed study. This is due to the fact that, in some cases, identification at species level is challenging, which can result in misidentification within the genus.

The genus *Sclerodermus* displays a strong consistency in its biological traits despite some interspecies variability. Developmental time is influenced by temperature, host size and foundress number across all species, with smaller hosts facilitating faster development and larger hosts supporting slower, extended development. These consistent patterns in temperature sensitivity and host utilisation reflect the genus’ adaptive strategies.

The relationship between brood size and host size shows a positive correlation across studies, and the number of foundresses significantly impacts offspring production and developmental time. As the number of foundresses increases, brood size rises, though per-foundress offspring production declines due to competition. The number of foundresses generally peaks at eight foundresses, beyond which offspring numbers remain stable, highlighting a limit to cooperative parasitism.

The analysis of offspring production in *Sclerodermus* species shows a significant variability based on the weight of the hosts and the number of foundresses. The study compiles data from 27 papers, offering a robust view of the dynamics influencing brood size. The offspring production in *Sclerodermus* species, as outlined in the data, shows a clear positive correlation between brood size and host size. This relationship, supported by previous studies [12,13,14,66], reinforces the notion that larger hosts provide more resources for the developing brood, leading to larger numbers of offspring. Similarly, the number of offspring produced by a single foundress also correlates positively with the size of the host, which is consistent with the ecological and evolutionary principles that larger resources and cooperative behaviour among parasitoids can enhance reproductive success [78,101]. However, it is important to note that while brood size increases with host size and foundress number, other factors like host health and environmental conditions may also play a role. The maximum offspring production by a single foundress, reported by [65] as 116, demonstrates the potential reproductive capacity of a *Sclerodermus* species, even in the absence of cooperative parasitism. This finding emphasises that, while cooperation among foundresses can significantly increase total brood size, a single female can still achieve considerable reproductive success, which could be an adaptive advantage in situations where host availability is limited or competition among foundresses is high.

Interestingly, eight studies lacked specific host weight data or had too much variability to be useful for detailed brood size analysis. These were categorised as “unknown weight”, limiting the ability to fully analyse the relationship between host size and brood production in those cases. This highlights a recurring challenge in such studies, where inconsistent reporting or wide variability in host characteristics can complicate comparative analysis. Standardising data collection practices across studies would greatly improve the reliability of meta-analyses.

The elevated reproductive success observed in multi-foundress systems indicates the potential benefits of social parasitism, whereby multiple females cooperatively exploit a single host. The decline in per-foundress productivity as group size increases mirrors classical patterns in gregarious parasitoids [11,68,69,70,71], where increased competition among foundresses leads to reduced fitness for each individual. Notably, one study [72] observed that beyond a certain “maximum” number of foundresses, brood production declines, possibly due to foundress competition or resource depletion. Another aspect to consider regarding competition in *Sclerodermus* is their occasional behaviour as hyperparasitoids. This behaviour has been observed for instances where *Sclerodermus* parasitises other parasitoid species, such as *Triaspis* sp. (Hymenoptera: Braconidae) [62] and even conspecifics [102]. These findings are consistent with studies on other gregarious parasitoids, where competition at high foundress densities reduces reproductive efficiency [103]. Furthermore, variability in offspring production relative to the number of foundresses and host size is a critical factor to consider in biological control programmes. This variability should be accounted for, at first, in the design of mass-rearing production systems within bio-farms and subsequently in determining the number of individuals required for effective biocontrol evaluations in field applications.

Overall, the data on offspring production in *Sclerodermus* spp. provide valuable insights into the reproductive strategies of these parasitoids. The strong correlation between host size, foundress number and brood size underscores the adaptability of *Sclerodermus* spp. to varying ecological conditions. Additionally, further investigation into the fitness outcomes for offspring in multi-foundress versus single-foundress systems, alongside studies on behaviour and competition among sibling and non-sibling individuals, could offer critical insights into social parasitism in *Sclerodermus* [104,105]. In this genus, multiple foundresses cooperate rather than compete, supporting each other to enhance reproductive success and survival. However, it remains unclear whether these foundresses operate as equals in a form of ‘democracy’ or if a hierarchy exists, with one female potentially assuming a dominant role [78,79]. Clarifying these social dynamics could deepen our understanding of the evolutionary pressures and adaptive behaviours underlying social parasitism in this group.

One of the more intriguing aspects of *Sclerodermus* reproduction is the highly female-biased sex ratio. This extreme bias, while typical of *Sclerodermus* spp., is unusual compared to other Hymenoptera parasitoids, which also show female-biased ratios but with a higher male percentage [11,12,13,15,25,31,41,63]. The persistence of such an unbalanced sex ratio gives rise to questions pertaining to evolutionary processes, as male offspring are a crucial component of future reproductive success. However, if the presence of males remains limited, it becomes challenging to ascertain the underlying advantage. The more recent hypotheses, such as dominance or infanticide proposed by some authors [79], offer plausible explanations, suggesting that intra-brood competition or behavioural dynamics among foundresses may drive the observed sex ratio.

The developmental time of *Sclerodermus* spp. is strongly influenced by multiple factors, primarily temperature and host species, as highlighted in numerous studies. Temperature is a key driver, as it accelerates or decelerates the developmental process depending on the range. Similar to many other insect species, *Sclerodermus* spp. showed faster development at higher temperatures (30 °C and 33 °C) across the studies, with developmental times extending significantly at lower temperatures like 21 °C and 20 °C [25,65,82,85]. This pattern underscores the essential role of temperature in insect metabolic processes [80,81]. Interestingly, despite species differences, developmental responses to temperature were remarkably consistent, with temperatures being distinctly grouped. This consistency is likely due to the geographic proximity of most species studied, suggesting similar environmental adaptations among these populations.

The host species also has a significant influence on the developmental rate of *Sclerodermus* females. Differences in the developmental rate have also been found in relation to the species analysed. The developmental rate of *Sclerodermus brevicornis* was found to be particularly rapid [33], whereas species such as *S. alternatusi* and *S. sichuanensis* developed more slowly, especially when reared on larger hosts [20,85], highlighting the interaction between host species and *Sclerodermus* species as a critical factor in developmental time. However, though developmental time and reproductive output are influenced by the number of foundresses, species and hosts, the genus maintains consistency in key traits.

Host size further contributes to developmental variability. Larger hosts provide more resources, allowing the parasitoid to develop over a longer period [18,92]. This trend suggests that parasitoids prioritising growth over speed tend to have longer developmental times when hosted by larger insects, as they have more reserves to exploit [24]. Such developmental flexibility allows *Sclerodermus* spp. to adapt to a wide range of host conditions.

Moreover, the differences in the timing of male and female development is consistent across the genus, as they are likely associated with differences in reproductive roles and energetic demands between the sexes [106]. Males have shorter lives than females and their emergence before females allows them to mate with females still in the cocoon. Additionally, the pre-ovipositional period varies based on temperature, host size and the number of foundresses. The period shortens as temperatures rise and as more foundresses are involved in parasitising a host [89,90]. Host size also plays a role, with larger hosts leading to longer pre-ovipositional periods, likely because the parasitoid needs more time to fully exploit the larger host before oviposition [18].

## 5. Conclusions

In conclusion, the genus *Sclerodermus* exhibits remarkable consistency in its biological and behavioural characteristics, despite some variability across species. While developmental times, host preferences and responses to environmental factors such as temperature and host size may vary among species, the overall patterns remain consistent. These parasitoids are highly adaptable, demonstrating similar ecological strategies in exploiting a wide range of host species and sizes, regardless of individual species differences.

Thus, even in the face of ecological and biological diversity, the genus *Sclerodermus* demonstrates a high degree of constancy in key life history traits. This consistency in developmental patterns, host exploitation and reproductive strategies highlights the genus’ resilience and adaptability, making it a valuable candidate for biological control efforts. The robustness of these characteristics across species within the genus further emphasises *Sclerodermus*’ evolutionary stability and effectiveness as a parasitoid across various ecological contexts.

## Figures and Tables

**Table 1:** Topic words used for literature classification.

Main Topics	Additional Topics
Behaviour	Antennal sensilla
Biological control	Biodiversity
Biology	Climate influence
Development	Field release
Distribution	Host influence
Mass-rearing	Host–parasitoid interaction
Medical	Interactions with other species
Molecular analysis	Learning
Systematics	Maternal care
Venom	Morphology
Odour cues	
	Parasitism rate
	Parasitoid–host ratio
	Proteins
	Reproduction
	Sequencing
	Sex ratio
	Sociality

**Table 2:** Mean time from egg to adult of females of *Sclerodermus* spp. at different temperatures (weighted by number of foundress). Significant differences (*p* < 0.05) are indicated by different letters, determined using Tukey’s post hoc test.

*Temp. (°C)*	*Mean Female Developmental Time (from Egg to Adult) ± SE (Days)*	*Number of Replicates*	*Sclerodermus Species*	*References*
20	36.1 ± 1.04 f	45	*S. puparie*; *Sclerodermus* sp.	[25,86]
21	54.5 ± 0.94 g	55	*S. puparie*; *S. alternatusi*	[65,85]
24	39.1 ± 0.90 f	55	*S. puparie*; *S. alternatusi*	[65,85]
25	30.5 ± 0.26 e	746	*S. puparie*; *S. brevicornis*; *S. alternatusi*; *S. sichuanensis*	[20,25,31,33,61,87,88]
25.5	28.3 ± 0.55 d	160	*S. guani*	[37]
26	26.2 ± 0.33 c	462	*S. brevicornis*; *S. harmandi*; *S. guani*	[15,62,63]
27	25.4 ± 0.94 bcd	55	*S. puparie*; *S. alternatusi*	[65,85]
30	21.80 ± 0.44 a	255	*S. puparie*; *S. alternatusi*	[25,65,82,85]
33	22.50 ± 0.94 ab	55	*S. puparie*; *S. alternatusi*	[65,85]

**Table 3:** Weighted mean (±SE) time from egg to adult of females of different species of *Sclerodermus* and different hosts (F_27, 501_ = 11.24, *p* < 0.001). Significant differences (*p* < 0.05) are indicated by different letters, determined using Tukey’s post hoc test.

*Sclerodermus Species*	*Host Species*	*N*	*Weighted Mean Time Development (from Egg to Adult) ± SE (Days)*
*S. alternatusi*	*Monochamus alternatus* Hope (Coleoptera: Cerambycidae)	277	32.30 ± 0.89 cd
	*Thyestilla gebleri* (Fald.) (Coleoptera: Cerambycidae)	360	34.00 ± 0.74 cd
*S. brevicornis*	*Psacothea hilaris hilaris* (Pascoe) (Coleoptera: Cerambycidae)	60	21.20 ± 1.80 ab
	*Anoplophora chinensis* (Forster) (Coleoptera: Cerambycidae)	32	24.00 ± 2.85 abcd
	*Corcyra cephalonica* (Stainton) (Lepidoptera: Gelechiidae)	195	25.80 ± 1.04 ab
	*Anoplophora glabripennis* (Motschulsky) (Coleoptera: Cerambycidae)	60	28.50 ± 1.81 abcd
*S. guani*	*Tenebrio molitor* L. (Coleoptera: Tenebrionidae)	625	26.30 ± 0.64 ab
*S. harmandi*	*Triaspis* sp. (Hymenoptera:Braconidae)	15	25.40 ± 2.96 abcd
	*Saperda populnea* (L.) (Coleoptera: Cerambycidae)	450	27.40 ± 1.28 abc
	*Monochamus alternatus* Hope (Coleoptera: Cerambycidae)	450	29.20 ± 1.26 abcd
	*Monochamus saltuarius* (Gebler) (Coleoptera: Cerambycidae)	-	26.6 ± 8.04 abcd
	*Psacothea hilaris* (Pascoe) (Coleoptera: Cerambycidae)	-	28.8 ± 8.04 abcd
*S. puparie*	*Agrilus planipennis* Fairmaire (Coleoptera: Buprestidae)	240	25.00 ± 0.74 a
	*Thyestilla gebleri* (Fald.) (Coleoptera: Cerambycidae)	334	26.80 ± 0.81 ab
	*Massicus raddei* (Blessig) (Coleoptera: Cerambycidae)	343	30.30 ± 0.80 bcd
	*Mesosa myops* (Dalman) (Coleoptera: Cerambycidae)	90	34.10 ± 1.21 cd
	*Moechotypa diphysis* (Pascoe) (Coleoptera: Cerambycidae)	90	34.70 ± 1.21 cd
	*Lamprodila virgata* (Motchulsky) (Coleoptera: Buprestidae)	60	37.50 ± 1.48 d
*S. sichuanensis*	*Sesamia inferens* (Walker) (Lepidoptera: Noctuidae)	15	33.50 ± 2.96 abcd
	*Bacchisa dioica* (Fairmaire, 1878) (Coleoptera: Cerambycidae)	15	33.80 ± 2.96 abcd
	*Callidium villosulum* Fairmaire (Coleoptera: Cerambycidae)	15	34.00 ± 2.96 abcd
	*Semanotus sinauster* Gressitt (Coleoptera: Cerambycidae)	150	34.40 ± 0.94 cd
	*Tenebrio molitor* L. (Coleoptera: Tenebrionidae)	15	34.70 ± 2.96 abcd
	*Dichocrocis punctiferalis* Guenée (Lepidoptera: Pyralidae)	15	34.70 ± 2.96 abcd
	*Ostrinia furnacalis* Guenée (Lepidoptera: Pyralidae)	15	34.90 ± 2.96 abcd
	*Chinolyda flagellicornis* (Smith) (Hymenoptera)	15	34.60 ± 2.96 abcd
*Sclerodermus* sp.	*Monochamus alternatus* Hope (Coleoptera: Cerambycidae)	30	32.50 ± 2.10 abcd

**Table 4:** Weighted (by the number of replicates) mean time (±SE) from egg to adult of males (offspring born from 1 foundress mother) of *Sclerodermus* spp. at different temperatures. Significant differences (*p* < 0.05) are indicated by different letters, as determined using Tukey’s post hoc test.

*Temp. (°C)*	*Mean Male Developmental Time (From Egg To Adult) ± SE (Days)*	*Number of Replicates*	*References*
21	61.9 ± 1.82 d	15	[65]
24	43.0 ± 1.82 c	15	[65]
25	25.0 ± 0.49 b	221	[18,20,61]
25.5	23.1 ± 0.79 ab	80	[34]
27	45.7 ± 1.05 c	45	[65,83]
30	20.4 ± 0.88 a	65	[65,82]
33	21.2 ± 1.83 ab	15	[65]

**Table 5:** Weighted mean pre-ovipositional time of *Sclerodermus* spp. at different temperatures. Significant differences (*p* < 0.05) are indicated by different letters, determined using Tukey’s post hoc test.

*Temp. (°C)*	*Weighted Mean Pre-Ovipositional Time ± SE (Days)*	*Number of Replicates*	*References*
21	12.67 ± 1.87 ab	6	[65]
24	9.25 ± 2.29 ab	4	[65]
25	10.65 ± 0.21 b	486	[38,61]
25.5	10.36 ± 0.36 b	160	[34]
26	6.81 ± 0.22 a	547	[63,89,95]
27	5.00 ± 2.65 ab	239	[65,83,96]
30	4.00 ± 2.65 ab	103	[65,82]
33	2.50 ± 3.24 ab	2	[65]

## Data Availability

Comprehensive literature and metadata can be downloaded from Malabusini, Serena and Lupi, Daniela (2024), “Unlocking *Sclerodermus* knowledge: a comprehensive database on this unique quasisocial genus of parasitoids”, Mendeley Data, V1, https://doi.org/10.17632/xxg8fx97xp.1 (accessed on 6 November 2024).
